# Peroral Amphotericin B Polymer Nanoparticles Lead to Comparable or Superior *In Vivo* Antifungal Activity to That of Intravenous Ambisome^®^ or Fungizone™

**DOI:** 10.1371/journal.pone.0025744

**Published:** 2011-10-06

**Authors:** Jagdishbhai L. Italia, Andrew Sharp, Katharine C. Carter, Peter Warn, M. N. V. Ravi Kumar

**Affiliations:** 1 Strathclyde Institute of Pharmacy and Biomedical Sciences, University of Strathclyde, Glasgow, United Kingdom; 2 School of Translational Medicine, The University of Manchester, Manchester, United Kingdom; Aristotle University of Thessaloniki, Greece

## Abstract

**Background:**

Despite advances in the treatment, the morbidity and mortality rate associated with invasive aspergillosis remains unacceptably high (70–90%) in immunocompromised patients. Amphotericin B (AMB), a polyene antibiotic with broad spectrum antifungal activity appears to be a choice of treatment but is available only as an intravenous formulation; development of an oral formulation would be beneficial as well as economical.

**Methodology:**

Poly(lactide-co-glycolode) (PLGA) nanoparticles encapsulating AMB (AMB-NPs) were developed for oral administration. The AMB-NPs were 113±20 nm in size with ∼70% entrapment efficiency at 30% AMB w/w of polymer. The *in vivo* therapeutic efficacy of oral AMB-NPs was evaluated in neutropenic murine models of disseminated and invasive pulmonary aspergillosis. AMB-NPs exhibited comparable or superior efficacy to that of Ambisome® or Fungizone™ administered parenterally indicating potential of NPs as carrier for oral delivery.

**Conclusions:**

The present investigation describes an efficient way of producing AMB-NPs with higher AMB pay-load and entrapment efficiency employing DMSO as solvent and ethanol as non-solvent. The developed oral formulation was highly efficacious in murine models of disseminated aspergillosis as well as an invasive pulmonary aspergillosis, which is refractory to treatment with IP Fungizone™and responds only modestly to AmBisome®.

## Introduction

The rate of opportunistic fungal pathogens causing life-threatening infections including aspergillosis, candidiasis and cryptococcosis are climbing inexorably, particularly within patients with cancer, organ transplant recipients, diabetics and the patients with congenital and acquired immunodeficiencies [1]–[3]. Invasive aspergillosis, in particular, is a leading cause of death in profoundly immunosuppressed patients [4]. Following environmental exposure to Aspergillus conidia, primary infection usually involves the respiratory tract. In severely immunocompromised patients, it may involve other organs, such as brain and sinus, or even cause disseminated infection [5]. Despite advances in the treatment, morbidity and mortality rate associated with invasive aspergillosis remains unacceptably high (70–90%) in immunocompromised patients [6].

AMB, a polyene antibiotic with broad spectrum antifungal activity, was also the therapy of choice for visceral leishmanisis [7], [8] but due to concerns about toxicity it is no longer the primary drug of choice for invasive fungal diseases [9]. AMB, due to its inherent low solubility and intestinal permeability, shows relatively poor oral bioavailability and hence is conventionally administrated parenterally as a miceller formulation with deoxycholate (Fungizone^TM^). However, conventional AMB therapy has limited efficacy in improving survival rate in conditions such as neutropenic patients with invasive aspergillosis due to a combination of poor efficacy and multiple toxicities including serious nephrotoxicity, haemolysis and liver damage as well as nausea and fever [10]–[11].

In order to improve the therapeutic index of AMB and reduce its associated toxicity, lipid-based formulations have been developed for parenteral administration, including (Ambisome®, Amphocil®, and Abelcet®). Despite the improvement in therapeutic index for lipid formulations of AMB, their use still remains limited due to higher cost, difficult route of administration and ongoing concerns about toxicity [9]. Therefore, the development of new, effective antifungal delivery systems remains an important intent.

The development of an effective oral formulation of AMB would have major applications in the treatment of invasive and disseminated fungal infections and would also dramatically expand access to the treatment of invasive mycosis and visceral leishmaniasis. Various formulations have been investigated for oral delivery of AMB including nanosuspensions and lipid-based formulations [12]–[14]. The nanosuspension of AMB exhibited improved solubility of AMB; however, was able to reduce the liver parasite burden only by 28.6% as compared to control in the murine model of visceral leishmaniasis indicating limited in vivo efficacy of the formulation [12]. The lipid-based oral formulations of AMB (Cochleates-based and Peceol®-poly(ethylene glycol)-phospholipids-based) formulations have been found to be effective in reducing tissue fungal burden in the murine models of disseminated aspergillosis and candidiasis [13], [14]; however, the formulation are yet to be evaluated in models of invasive aspergillosis model, which is more refractory to treatment and difficult to treat. We have recently reported the improved oral bioavailability and reduced nephrotoxicity of biodegradable nanoparticles encapsulating AMB in rodents [15]. The present study highlights further optimization of the formulation and its efficacy in murine models of disseminated and invasive pulmonary aspergillosis following oral administration.

## Results and Discussion

### Preparation and optimization of nanoparticles

#### The choice of non-solvent

**Figure 1:**
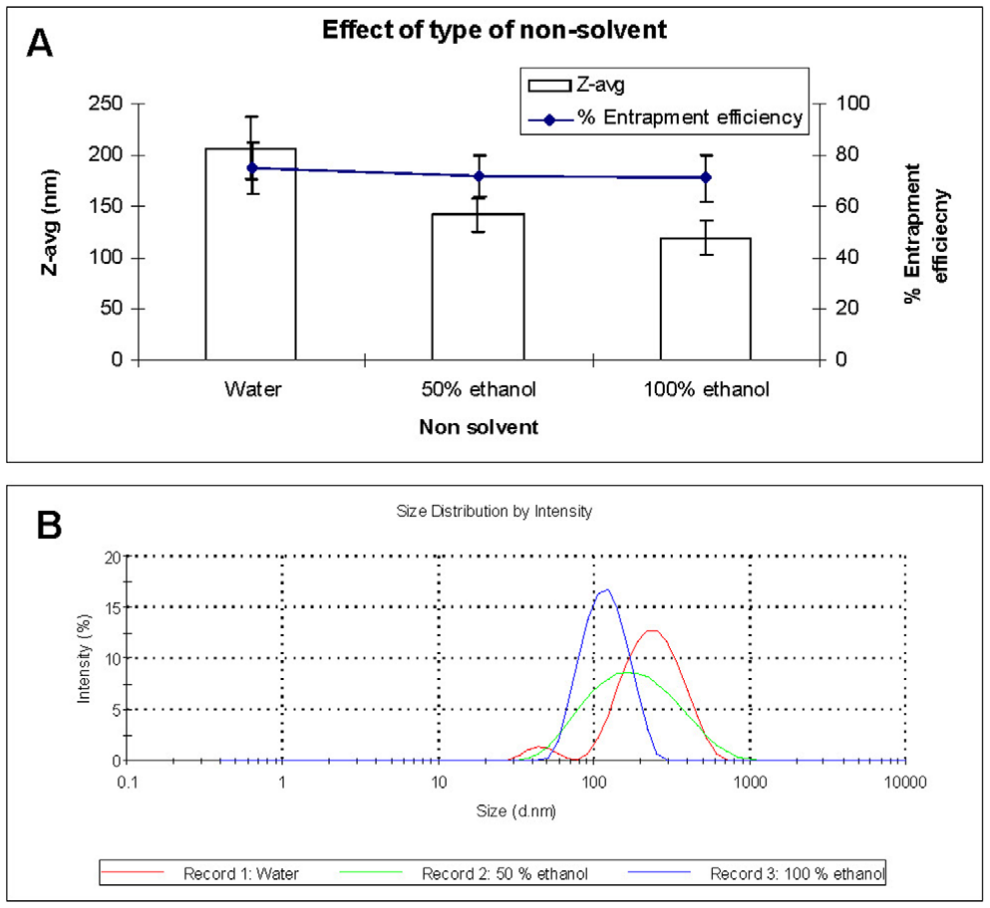
The effect of non-solvent composition on particle size, entrapment efficiency (A) and particle size distribution (B). PDI, water: 0.3±0.1; 50% ethanol 0.1±0.07; ethanol 0.1±0.02.

The mean particle size was clearly dependent on the type of the non-solvent used and followed the order water>50% ethanol>100% ethanol (Fig. 1A). Ethanol is presumably a “poorer” solvent for PLGA as compared to water, and it promotes the precipitation of the polymer more actively [16], [17]. The size distribution was also changed considerably with the change in the non-solvent composition, where particles made with 100% ethanol show narrow distribution profile (Fig. 1B). Since, the volume of DMSO remained constant, no considerable change was observed in the entrapment efficiency (Fig. 1A).

The addition of a surfactant (VE-TPGS) was necessary for stabilization of AMB-NPs suspension as aggregation was observed without use of VE-TPGS. Considering the lowest particle size and narrow size distribution, 100% ethanol was used as non-solvent for the subsequent experiments.

#### The solvent composition (DMSO:Acetone)

**Figure 2:**
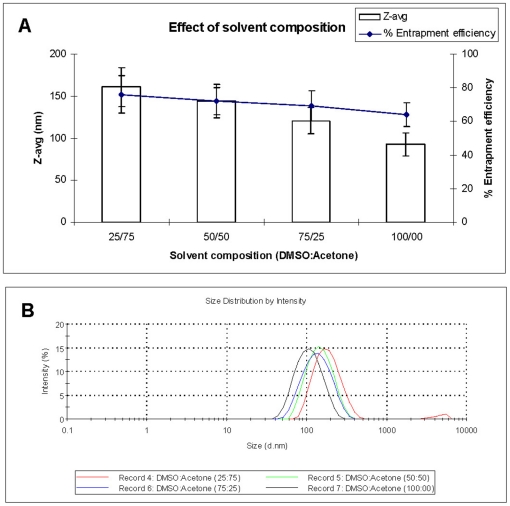
The effect of solvent composition (DMSO:acetone) on particle size, entrapment efficiency (A) and particle size distribution (B). For all the preparations, PDI is 0.1±0.01.

The particle characteristics were significantly affected by the composition of organic solvent. The particle size as well as entrapment efficiency were decreased (160±23 to 93±14 nm and 76±11 to 64±7%, respectively) as the fraction of DMSO increased in the solvent mixture (DMSO:acetone, 25∶75>50∶50>75∶25>100∶00) with little change in the size distribution pattern (Fig 2A&B). The smallest particles were obtained with the use of 100% DMSO as the solvent. The trend of decrease in particle size with increase in the DMSO fraction can be explained by the physico-chemical properties of the solvents and non-solvents used in the nanoparticles preparation. The property parameters of the solvents and non-solvents including the polarity, donicity, and the acceptivity can be quantitatively characterized by solvent polarity/polarizability, solvent basicity, and solvent acidity values, respectively. All of the solvents or non-solvents used in the present study can be classified into three types: (a) type I, strong electron pair donor (EPD) with high polarity, high basicity, and low acidity; (b) type II, solvents with medium polarity and low acidity; (c) type III, solvents with strong electron pair accepter (EPA) with medium polarity and high acidity. DMSO (type I) is a good electron pair donor (EPD) solvent, with high basicity due to the presence of its lone electron pairs [18]. Acetone (type II) is a solvent with no EPD or EPA activity and having low acidity. Ethanol (type III) is a good EPA nonsolvent with high acidity. Therefore, a *strong* EPD-EPA interaction arises between DMSO and ethanol molecules and thus DMSO molecules interacts with the ethanol molecules with higher affinity as compared to acetone molecules, which could lead to faster diffusion of the DMSO in the ethanol as compared to acetone and thus smaller particle size [19]. Since AMB has very good solubility in the DMSO as compared to acetone, there was a little decrease in the entrapment efficiency with increase in the DMSO fraction in the solvent mixture (Fig 2A). Considering smaller particle size, 100% DMSO was used as solvent for further experiments.

#### The solvent volume

**Figure 3:**
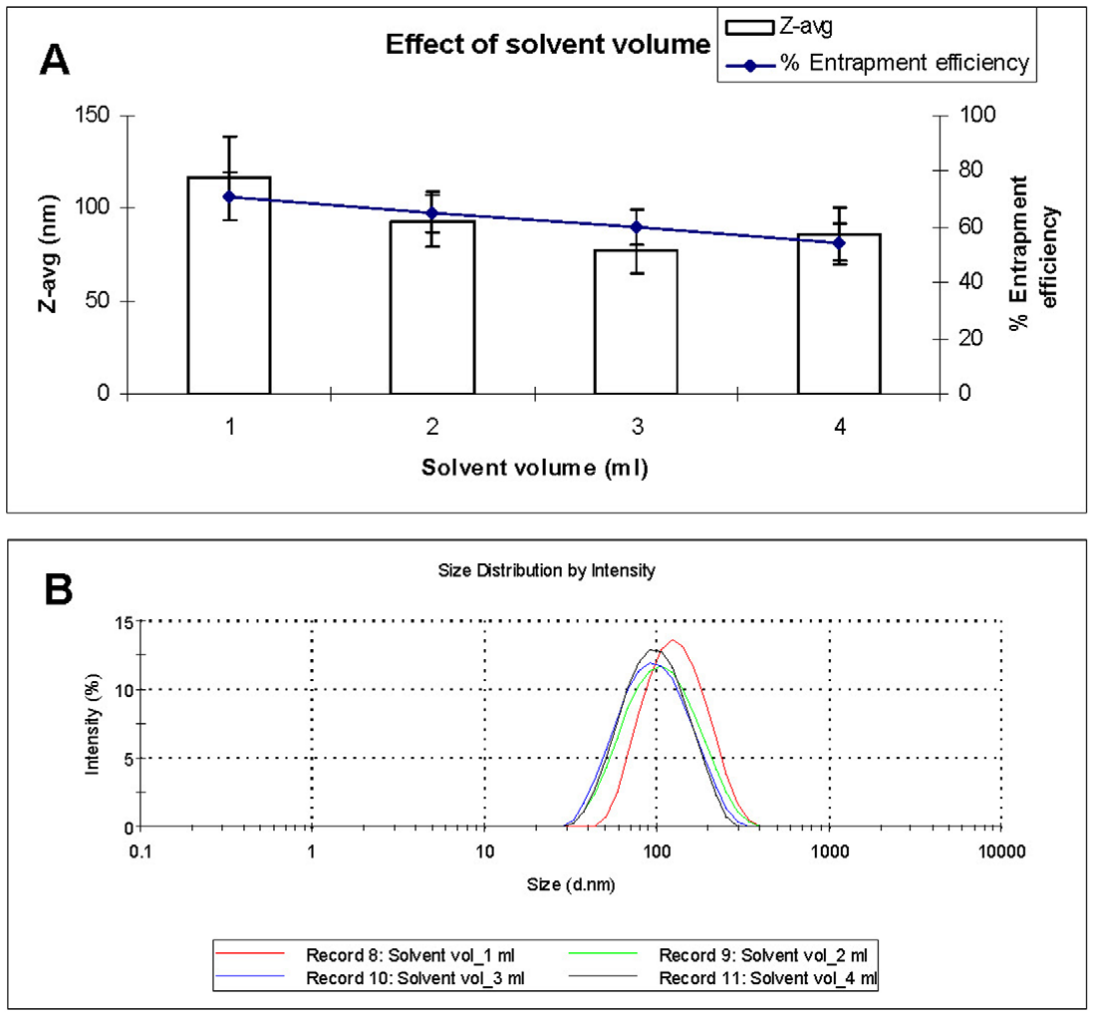
The effect of solvent (DMSO) volume on particle size, entrapment efficiency (A) and particle size distribution (B). For all the preparations, PDI is 0.1±0.01.

Four volumes of DMSO (1, 2, 3 and 4 ml) were used to prepare AMB-NPs and its effect on particle size, size distribution and entrapment efficiency was evaluated. With increasing solvent volume from 1 to 4 ml, there was a decrease in particle size (116±22 to 86±14 nm) and entrapment efficiency (71±9 to 54±7%) was observed (Fig 3A & B). This trend might be explained by considering two facts: (i) the number of polymer chains per unit volume of solvent and (ii) the influence of polymer concentration on the viscosity. First with the lowest volume of solvent, there are greater number of polymer chains per unit volume of solvent and as a consequence of the solvent diffusing into the non-solvent carries out more polymer chains which aggregate and thus form larger particle. This phenomenon is also favoured by the fact that increasing polymer concentration increases polymer-polymer interactions which means that more polymer chains remain associated during the diffusion process. On the other hand, the influence of polymer concentration on the viscosity of the organic phase is also taken into account. On increasing the polymer concentration, a more viscous organic phase is obtained, which provides a higher mass transfer resistance; thus the diffusion of polymer-solvent phase into the external aqueous phase is reduced and larger NP are formed [19]. In contrast, a diminution in the polymer concentration (due to increase in the solvent volume) decreases the viscosity of the organic phase, which increases the distribution efficiency of the polymer-solvent phase into the non-solvent leading to formation of smaller particles.

The decrease in AMB entrapment could be due to increase in solubility of AMB in external phase due to increase in DMSO volume. Considering the particle size and entrapment efficiency, 3 ml of organic solvent was selected for further studies.

#### The initial AMB loading

**Figure 4:**
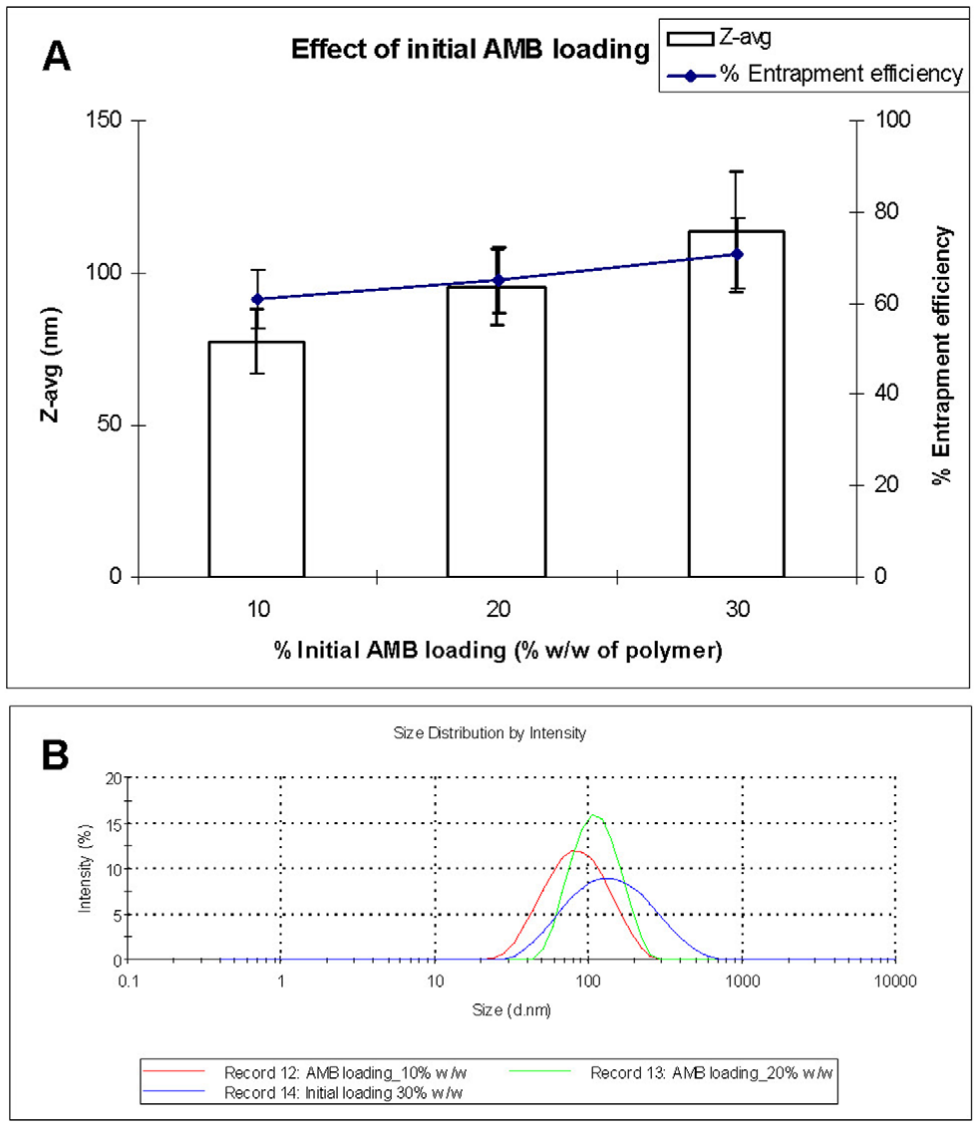
The effect of initial AMB loading on particle size, entrapment efficiency (A) and particle size distribution (B). PDI for 10% Loading: 0.1±0.01; 20%: 0.2±0.00; 30%: 0.2±0.01.

Effect of initial AMB loading on particle size, size distribution and entrapment efficiency was evaluated using four different pay loads (10, 20, 30 and 40% w/w of polymer). With increasing initial loading from 10 to 30% w/w, an increase in the particle size (77±10 to 113±15 nm) and entrapment efficiency (61±6 to 71±9) was observed with widening of the size distribution (Fig. 4A&B). The increase in the particle size could be due to the increase in the viscosity of the organic phase with resultant slower diffusion of the solvent into non-solvent and higher particle size. Above 30% w/w loading, the precipitation was observed indicating that PLGA could not hold AMB above this concentration. The formulation with 30% w/w of AMB loading was selected for the *in vivo* studies.

### Efficacy of AMB-NPs in murine models of pulmonary and disseminated aspergillosis

#### Inhalational murine model of invasive pulmonary aspergillosis

**Figure 5:**
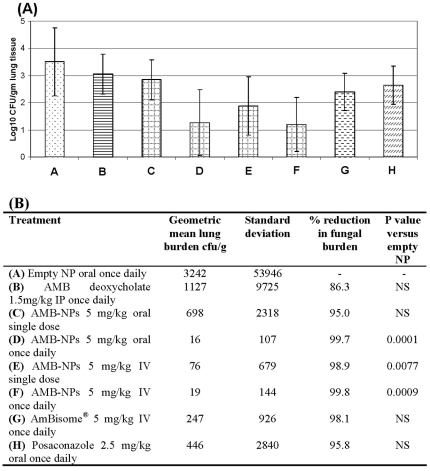
Lung burden of mice with invasive pulmonary aspergillosis treated with AMB-NPs, AMB deoxycholate, AmBisome and Posaconazole 101 hours post infection (Panel A). Geometric mean tissue burdens (and standard deviations) of mice with invasive pulmonary aspergillosis treated with AMB-NPs, AMB deoxycholate, AmBisome and Posaconazole 101 hours post infection (**Panel B**). The p values are from a Kruskal Wallis test (Conover-Inman).

Control mice developed a heavy lung burden that was associated with weight loss and respiratory distress. Treatment with 1.5 mg/kg/day FungizoneTM IP, 5 mg/kg/day AmBisome® IV, 2.5 mg/kg/day posaconazole oral and the single dose of 5mg/kg oral AMB-NPs caused numerical reductions in tissue burden none of which were statistically significant (p>0.05) **(** Fig. 5 **)**. Treatment with 5 mg/kg AMB-NPs either administered as single dose IV (p = 0.0077) or daily dosing IV (p = 0.0009) or orally caused large statistically significant reductions in lung burden. Of note AMB-NPs when administered as a single 5 mg/kg IV dose was superior to 1.5mg/kg/day conventional AMB (p = 0.0156) similarly AMB-NPs 5 mg/kg/day were also superior regardless of whether the drug was administered orally (p = 0.002) or IV (p = 0.0003).

#### Murine model of disseminated aspergillosis

In this model control mice developed heavy and reproducible kidney burdens but remained healthy throughout the experimental observation period. Treatment with FungizoneTM IP (0.1 or 0.3 mg/kg/day), single oral doses of 2 or 5 mg/kg AMB-NPs, 0.3 mg/kg/day IV AMB-NPs, and 2.5 mg/kg/day oral posaconazole all significantly reduced the kidney fungal burden. Numerically treatment with 0.3 mg/kg/day AMB-NPs was superior to FungizoneTM but the difference was not statistically significant.

**Figure 6:**
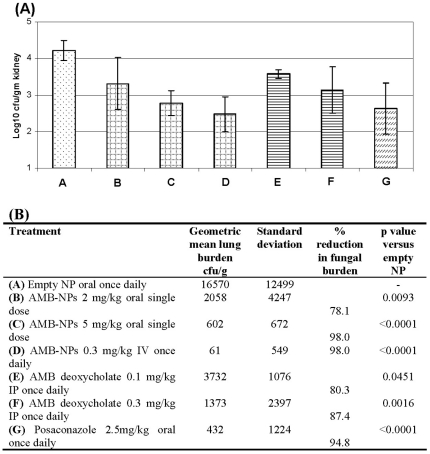
Kidney burden of mice with disseminated aspergillosis treated with AMB-NPs, AMB deoxycholate, and Posaconazole 101 hours post infection (Panel A). Geometric mean tissue burdens (and standard deviations) of mice with disseminated aspergillosis treated with AMB-NPs, AMB deoxycholate, and Posaconazole 101 hours post infection (**Panel B**). The p values are from a Kruskal Wallis test (Conover-Inman).

Delmas *et al.,* (2002) reported AMB-Cochleates formulation with significant anti-fungal activity upon oral administration. The formulation at the total dose of 560 mg/kg (administered in divided doses as 40 mg/kg daily for 14 days) caused 2 log reduction in fungal burdens of lungs, livers and kidneys of the neutropenic mice infected with *A. fumigatus* [13]. Considering the dose and duration of the therapy, the efficacy of the formulation was only moderate. In present studies, oral AMB-NPs exhibited better therapeutic efficacy and caused 2.14 logs reduction in lung fungal burden in invasive model with just 20 mg/kg total dose (administered at 5 mg/kg daily for 4 days) (Fig. 5A). Similar efficacy was observed in the disseminated model where oral AMB-NPs caused 1.44 logs reduction in kidney fungal burden following single oral dose of 5 mg/kg (Fig. 6A).

Risovic *et al.,* (2007) evaluated the anti-fungal efficacy of an oral lipid based formulation of AMB made with Peceol® in male albino Sprague-Dawley rats infected with *A. fumigatus*. Treatment with Peceol-AMB at the dose of 50 mg/kg/day for 4 days significantly reduced fungal burdens only in the brain and spleen but had no effect on fungal burdens in the kidneys, heart, liver or lungs of treated animals compared to control values [20]. Recently, Wasan *et al.,* (2009) determined the anti-fungal efficacy of a Peceol/distearoylphosphatidylethanolamine-poly(ethylene glycol)2000 based oral formulation of AMB in immunosuppressed male albino Sprague–Dawley rats infected intravenously with the *A. fumigatus*. Oral administration of the formulation at a total dose of 40 mg/kg (administered in 4 divided doses, 10 mg/kg twice a day for 2 days) resulted in ∼95% suppression of kidney fugal burdens compared to controls [14]. AMB-NPs showed better efficacy and suppressed kidney fungal burden by >97% following oral treatment at just 5 mg/kg single dose (Fig. 6-Panel B). Overall, oral treatment with AMB-NPs as either a single or multiple dose exhibited anti-fungal activity comparable/better to parenteral therapy with FungizoneTM and AmBisome®. Considering shorter duration of therapy in the present experiment, the longer dosage regimen (as used in human) should completely cure the infection. Suitability of AMB-NPs for intravenous bolus injection presents additional advantage, offering possibility of alternate dosage form, which could provide convenience in switching between intravenous and oral formulations.

The IPA model used in the present study is a very severe model and the therapy with conventional AMB is not very effective at treating this model. In the present study, the oral and intravenously administered AMB-NPs were highly effective for treating the infected animals. The impressive oral efficacy of the AMB-NPs could be the result of enhanced oral absorption. Nanoparticles are known to enhance the intestinal uptake of the encapsulated drugs by various mechanisms including protecting drug from degradation in harsh gastro-intestinal pH and enzymes, bypassing first pass metabolism, and increasing the lymphatic uptake [21]–[23]. Thus, improved efficacy of AMB-NPs could be attributed to enhanced oral bioavailability. The present experiments illustrate that oral AMB-NPs are effective in treating disseminated and invasive aspergillosis indicating that NPs are promising carriers for the oral delivery of AMB.

## Materials and Methods

### Preparation and optimization of AMB-NPs

AMB-NPs were prepared by adapting a method developed in our laboratory with appropriate modifications [**15**]. Briefly, 5 mg of AMB (Fluorochem Ltd, UK) and 50 mg of PLGA (Resomer RG 50∶50 H; inherent viscosity 0.41 dl/g) (Boehringer, Ingelheim, Germany) were dissolved in a suitable organic solvent (S) either DMSO (Fisher Scientific Loughborough UK) or mixture of DMSO and acetone (Fisher Scientific). This phase was then added to the dispersing phase (10 ml of water or 50% v/v ethanol (Sigma Aldrich, Poole, UK) or 100% ethanol) under moderate magnetic stirring. The dispersing phase comprises a liquid in which the polymer is insoluble-the non-solvent (NS)-containing a surfactant Vitamin E-TPGS (VE-TPGS), which was a gift sample from Eastman Chemical Company (Llangefni, UK). The preparation was then kept overnight in a fume hood under stirring to allow evaporation of ethanol. The particles were then purified by ultrafiltration and freeze-dried using 10% w/v of sucrose a as cryoprotectant. *The method was successful at 1 gm batch size with excellent reproducibility with respect to particle size, entrapment efficiency and most importantly recovery.*

The size of AMB-NPs was determined by dynamic light scattering (NanoZS, Malvern Instruments, UK) as an average of 5 measurements. The amount of the drug entrapped in AMB-NPs was determined by dissolving known amount of formulation in DMSO followed by appropriate dilution with methanol and analysis was performed by a validated reverse phase high performance liquid chromatographic (RP-HPLC) method [15]. The nanoparticulate formulation was thoroughly optimized for particle size and entrapment efficiency by optimization of composition of non-solvent, composition and volume of organic solvent and initial AMB loading.

#### Selection of the ‘non-solvent’

The effect of type of non-solvent (water, 50% ethanol and 100% ethanol) on particle size, size distribution and entrapment efficiency was studied. Briefly, 5 mg of PLGA was dissolved in 0.65 ml of DMSO and 50 mg of PLGA was dissolved in 2 ml of Acetone. The PLGA solution was then slowly added to the DMSO solution containing AMB under stirring. The drug-polymer solution was then drop-wise added into 10 ml of non-solvent, either water, 50% ethanol or 100% ethanol containing 1.4% w/v of VE-TPGS under stirring.

#### Determining the solvent composition (DMSO:Acetone)

The nanoparticles were prepared with various compositions of organic solvents (DMSO:acetone 25∶75, 50∶50, 75∶25 and 100∶00) and its effect on the particle characteristics studied. All other parameters (PLGA, 50 mg; AMB, 5 mg; organic solvent, 2 ml of DMSO; non-solvent, 10 ml of ethanol containing 1 mg/ml of VE-TPGS) were kept constant.

#### Effect of solvent volume

The effect of volume of DMSO on particle size, size distribution and entrapment efficiency was determined. The volumes studied were 1, 2, 3 and 4 ml of DMSO. All other parameters (PLGA, 50 mg; AMB, 5 mg; non-solvent, 10 ml of ethanol containing 1 mg/ml of VE-TPGS) were kept constant.

#### Effect of initial loading of Amphotericin B

The influence of initial AMB loading on particle characteristics was studied using 10, 20, 30 and 50% w/w of polymer. All other parameters (PLGA, 50 mg; DMSO volume, 3 ml and non-solvent, 10 ml of ethanol containing 1 mg/ml of VE-TPGS) were kept constant.

### Efficacy of AMB-NPs in murine models of pulmonary and disseminated aspergillosis

All animal experiments included in this study were part of ongoing studies performed under UK Home Office project licence PPL40/3101 and had received ethical clearance from the University of Manchester Ethics Review Panel. Male CD1 mice (Charles River Ltd., Kent, United Kingdom) weighing 22 to 25 g were used. The mice were housed in vented HEPA-filtered cages, and food and water were provided *ad libitum*.

### Inhalational murine model of invasive pulmonary aspergillosis

A persistently neutropenic inhalational murine model of invasive pulmonary aspergillosis was used to examine the in vivo efficacy of AMB-NPs. Briefly, mice were rendered neutropenic on day minus 2 and +3 with; cyclophosphamide (Baxter Healthcare ltd, Norfolk, UK) 200 mg/kg intraperitoneally (IP) and cortisone acetate (Sigma) 250 mg/kg subcutaneously (SC), which resulted in profound and persistent neutropenia for 6 days. Mice (n = 6 per cage) were exposed to 12 mL of a suspension containing 8.1 x 108 A. fumigatus A1163 spores/mL, harvested from 7 day old cultures on Sabouraud glucose agar (Oxoid Basingstoke UK) by flooding the plate with phosphate buffered saline (Invitrogen, Paisley, UK) with 0.05% Tween 80 (Sigma), that was nebulized (Hudson RCI, High Wycombe, UK) at 1 bar for one hour. The desired inoculum was verified by quantitative culture [24].

AMB-NPs were diluted in saline and administered at 5 mg/kg both IV and by oral gavage. AMB-NPs were either administered once 5 hours post treatment or once daily. AMB (FungizoneTM ER Squibb & Sons Ltd, Hounslow, England) was diluted to the desired concentration in 5% glucose and was administered IP once daily at 1.5 mg/kg. AmBisome® (Gilead Sciences Int, UK) was diluted in 5% glucose administered once daily IV at 5 mg/kg. Posaconazole (Noxafil® Schering) was diluted in 20% (2-Hydroxypropyl)-β-cyclodextrin (Sigma) and administered at 2.5 mg/kg once daily by oral gavage. Control mice were administered empty nanoparticles IV once daily. Therapy was initiated 5-hours post-exposure and was continued for 4 days (4 doses for most drug). Mice were euthanized 101 hours post infection and the lungs cultured quantitatively on Sabouraud dextrose agar.

### Murine model of disseminated aspergillosis

A temporarily neutropenic murine model of disseminated aspergillosis was also used to examine the *in vivo* efficacy of AMB-NPs. Briefly, mice were rendered neutropenic on day minus 3 with; cyclophosphamide 200 mg/kg intraperitoneally (i.p.) which resulted in profound neutropenia for 3 days post infection. Mice (n = 5 per cage) were infected with 8 x 104 *A. fumigatus* A1163 spores per 25 g mouse on day 0 via the lateral tail vein (3 days post immunosuppression). The desired inoculum was verified by quantitative culture [25].

Mice were treated as above 2 and 5 mg/kg AMB-NPs once only by oral gavage, 0.3 mg/kg AMB-NPs IV once daily, Posaconazole 2.5 mg/kg oral once daily or control (empty nanoparticles) IV once daily. Therapy was initiated 5-hours post-exposure and was continued for 4 days (4 doses for all drugs other than AMB-NPs). Mice were euthanized 101 hours post infection and the kidneys cultured quantitatively on Sabouraud dextrose agar.

Statistics. In the present studies, normality of the data was first analysed using Minitab 15 statistical software using Kolmogorov-Smornov Test. Since the data was found to be non-normally distributed, Kruskal Wallis test (non-parametric equivalent test of ANOVA) was applied.
